# The Relationship Among Intra-Amniotic Inflammatory Response, The Progression of Inflammation in Chorionic Plate and Early-Onset Neonatal Sepsis

**DOI:** 10.3389/fped.2021.582472

**Published:** 2021-04-29

**Authors:** Kyung Chul Moon, Jeong-Won Oh, Chan-Wook Park, Joong Shin Park, Jong Kwan Jun

**Affiliations:** ^1^Department of Pathology, Seoul National University College of Medicine, Seoul, South Korea; ^2^Department of Obstetrics and Gynecology, Soonchunhyang University Seoul Hospital, Seoul, South Korea; ^3^Department of Obstetrics and Gynecology, Seoul National University College of Medicine, Seoul, South Korea; ^4^Seoul National University Medical Research Center, Institute of Reproductive Medicine and Population, Seoul, South Korea

**Keywords:** ascending intra-uterine infection, chorionic plate inflammation, early-onset neonatal sepsis, intra-amniotic inflammatory response, preterm birth

## Abstract

**Background:** The chorionic plate (CP) has been denigrated by the well-known route of the extraplacental membranes from the decidua parietalis through the chorion to the amnion in the progression of ascending intrauterine infection among preterm births (PTBs). However, considering previous studies reporting the relationship among intra-amniotic inflammatory response (IAIR), the progression of inflammation in extraplacental membranes and early-onset neonatal sepsis (EONS), and the anatomic connection between extraplacental membranes and CP, there is a good chance that IAIR would be more likely and severe according to the progression of inflammation in CP, and this progression of inflammation in CP would be associated with a significant increase in EONS in neonates delivered due to either PTL or preterm-PROM. Unfortunately, there is no information about the relationship among IAIR, the progression of inflammation in CP, and EONS among spontaneous PTBs. The objective of the current study is to examine this issue.

**Method:** The study population included 309 singleton pregnant women-delivered preterm neonates with the following conditions: (1) gestational age (GA) at delivery: 20.0~36.9 weeks; (2) spontaneous PTBs: PTL (151 cases) or preterm-PROM (158 cases); (3) available results of placental histologic examination; (4) without congenital anomaly; and (5) delivery within 60 h of amniocentesis. We examined IAIR, and the frequency of intra-amniotic inflammation (IAI) and EONS according to the progression of inflammation in CP [i.e., stage-0, inflammation-free CP; stage-1, inflammation restricted to subchorionic fibrin (SCF); stage-2, inflammation in connective tissue (CT) of CP but without chorionic vasculitis; and stage-3, chorionic vasculitis]. IAIR was determined by amniotic fluid (AF) matrix metalloproteinase-8 (MMP-8) concentration (ng/ml), and IAI was defined as an elevated AF MMP-8 concentration (≥23 ng/ml). EONS included either suspected or proven EONS.

**Results:** (1) Each stage (stage-0 to stage-3) was present in 69.3% (214/309), 15.9% (49/309), 11.0% (34/309), and 3.9% (12/309) of the study population. (2) AF MMP-8 concentrations continuously elevated according to the progression of inflammation in CP [stage-0 vs. stage-1 vs. stage-2 vs. stage-3; median (ng/ml), range (ng/ml); 6.0 (0.3–4202.7) vs. 153.9 (0.3–6142.6) vs. 464.9 (5.8–3929.0) vs. 1,780.4 (35.1–5019.5); Kruskal–Wallis test, *P* < 0.001 and Spearman's rank-correlation test, *P* < 0.000001, *r* = 0.553]. (3) Moreover, the frequency of IAI and EONS gradually increased with the progression of inflammation in CP [stage-0 vs. stage-1 vs. stage-2 vs. stage-3; IAI, 30.5% (64/210) vs. 70.2% (33/47) vs. 96.7% (29/30) vs. 100% (12/12); EONS, 3.5% (7/200) vs. 25.5% (12/47) vs. 32.3% (10/31) vs. 40.0% (4/10); each for Pearson's chi-square test, *P* < 0.000001 and linear-by-linear association, *P* < 0.000001]. (4) Of note, multiple logistic regression analysis demonstrated that a more advanced stage in the progression of inflammation within CP was associated with a higher odds ratio (OR) for EONS [stage-1 vs. stage-2 vs. stage-3; OR, 7.215, 95% confidence-interval (CI) (2.177–23.908) vs. OR, 10.705, 95% CI (2.613–43.849) vs. OR, 27.189, 95% CI (2.557–289.124)] compared with stage-0 even after the adjustment for potential confounding variables.

**Conclusion:** IAIR is more likely and severe according to the progression of inflammation in CP, and this progression of inflammation in CP is an independent risk factor for EONS in spontaneous PTBs. This finding suggests that CP may be another playground for the progression of ascending intrauterine infection in addition to extraplacental membranes, and the progression of inflammation in CP may be used for the prediction of EONS in spontaneous PTBs.

## Introduction

The causes of spontaneous preterm birth (PTB) remain largely elusive (1). Ascending intrauterine infection is one of the causes of spontaneous PTB (1, 2). Ascending intrauterine infection proceeds from the vagina–cervical canal through the chorio-decidua to either the chorionic vessels (CVs) or the amnion and amniotic cavity, and finally invades the fetus (2). Moreover, ascending intrauterine infection evokes inflammatory responses in each placental compartment [i.e., extraplacental membranes, chorionic plate (CP), and umbilical cord] (3–7) and amniotic fluid (AF) (8, 9). During the progression of ascending intrauterine infection in extraplacental membranes, maternal neutrophils migrate from the decidua parietalis *via* the membranous trophoblast and connective tissue (CT) of the chorion to the amnion in an outside-in pattern within the extraplacental membranes, and this progression of inflammation within the extraplacental membranes is associated with more frequent positive AF culture and more intense intra-amniotic inflammatory response (IAIR) (10–12).

Extraplacental membranes and CP have a similarity of structural arrangement in that the membranous trophoblast and CT of chorion in the extraplacental membranes locate between the decidua parietalis and the amnion, and the subchorionic fibrin (SCF) and CT in CP lie between the decidua basalis/inter-villous space (IVS) and the amnion. Moreover, the extraplacental membranes are anatomically connected with the CP layer by layer as in the following: (1) the decidua parietalis in the extraplacental membranes is attached to the decidua basalis beneath the placental disc; (2) the membranous trophoblast of the chorion in the extraplacental membranes is connected with the SCF in CP; (3) the CT of the chorion in the extraplacental membranes is directly linked to the CT in CP; and (4) the amnion covers the innermost part of both the extraplacental membranes and CP.

Considering the previous studies reporting the relationship among IAIR, the progression of inflammation in the extraplacental membranes and early-onset neonatal sepsis (EONS) (10–12), and the anatomic connection between the extraplacental membranes and CP, there is a good chance that IAIR would be more likely and severe according to the progression of inflammation in CP, and this progression of inflammation in CP would be associated with a significant increase in EONS in neonates delivered due to either preterm labor with intact membranes (PTL) or preterm premature rupture of membranes (preterm-PROM). Unfortunately, there is a paucity of data about the relationship among IAIR, the progression of inflammation in CP, and EONS among spontaneous PTBs (Supplementary Table 1) (4, 13–23). Therefore, we performed the current study to examine this issue.

## Materials and Methods

### Study Design and Patient Population

Study population included 309 singleton pregnant women-delivered preterm neonates at the Seoul National University Hospital from February 1993 to April 2004. Patients were eligible for inclusion if they met the following conditions: (1) gestational age (GA) at delivery between 20.0 and 36.9 weeks, (2) types of preterm birth: PTL (151 cases), or preterm-PROM (158 cases), (3) available results of placental histologic examination after delivery, (4) without anomaly, and (5) delivery within 60 h of amniocentesis (Supplementary Figure 1). The criterion of amniocentesis-to-delivery interval was applied to preserve a meaningful temporal relationship between the results of AF studies and the pathologic findings of placenta obtained at delivery. At our institution, trans-abdominal amniocentesis for retrieval of AF in order to identify microbiologic and inflammatory status with fetal lung maturity and placental pathologic examination after delivery were routinely recommended and performed on all pregnant women hospitalized with either PTL or preterm-PROM. PTL and preterm-PROM were diagnosed with previously published criteria (24, 25). The intensity of IAIR, and the frequency of intra-amniotic inflammation (IAI) and positive AF culture were studied according to the progression of inflammation in CP. Written informed consent was acquired from the study population. The Institutional Review Board of our institute (SNUH) specifically approved the current study (IRB No. 1909-128-1066).

### Clinical Characteristics and Pregnancy Outcomes Including Early-Onset Neonatal Sepsis

The clinical characteristics of mothers and their neonates were investigated from the medical records. Data included maternal age, parity, GA at amniocentesis, type of preterm delivery, GA at delivery, birth weight, gender of newborn, 1- and 5-min Apgar scores, delivery mode, the frequency of inflammation in extraplacental membranes (i.e., chorio-deciduitis and amnionitis) and funisitis, and antenatal corticosteroids and antibiotic use. EONS was proved in the presence of a positive blood culture result within 72 h of delivery and was suspected in the absence of a positive culture when two or more items were obtained among the following items: (1) WBC <5,000 cells/mm^3^, (2) polymorpholeukocytes <1,800 cells/mm^3^, and (3) band neutrophils/total neutrophils > 0.2. These diagnostic criteria were previously used in the literature (10, 26, 27). EONS was defined in the presence of proven or suspected EONS in the current study. We excluded 21 neonates in the analysis of EONS due to extremely premature birth and subsequent immediate death (11 cases) or no information about EONS in the medical records (10 cases) and thus could not evaluate the presence or absence of EONS in them.

### Placental Pathologic Examination

Placental tissue samples for pathologic examination were extraplacental membranes, CP, and umbilical cord. Formalin-fixed paraffin-embedded (FFPE) blocks with placental tissue samples were made and stained with hematoxylin and eosin (H & E). Clinical information was not supplied to pathologists. Chorio-deciduitis, amnionitis, and funisitis were diagnosed according to the criteria previously defined (14) as in the following; (1) for chorio-deciduitis, at least one focus of more than five neutrophils in the chorio-decidua; (2) for amnionitis, at least one focus of more than five neutrophils in the amnion; and (3) for funisitis, neutrophil infiltration into the umbilical vessel walls or Wharton's jelly. Inflammation in CP was diagnosed as neutrophilic infiltration in CP with the use of the previously reported criteria (14), and the progression of inflammation in CP was classified into four stages with a slightly modified criteria from a previous report (28) (i.e., stage 0, inflammation-free CP; stage 1, inflammation restricted to SCF; stage 2, inflammation in the CT of CP without chorionic vasculitis; and stage 3, chorionic vasculitis).

### Amniotic Fluid Studies

AF was cultured for the evaluation of microbial invasion to the amniotic cavity (i.e., aerobic and anaerobic bacteria, and genital mycoplasmas such as *Ureaplasma urelyticum* and *Mycoplasma hominis*) according to the methods previously described (24, 25). The remaining fluid was centrifuged and stored in polypropylene tubes at −70°C (29). Matrix metalloproteinase-8 (MMP-8) concentrations in stored AF were measured with a commercially available enzyme-linked immunosorbent assay (Amersham Pharmacia Biotech, Inc., Little Chalfont, Bucks) (29). Details about this assay and its performance have been previously reported (29). The intensity of IAIR was gauged by AF MMP-8 level (ng/ml), and IAI was defined as the presence of an elevated AF MMP-8 level (≥23 ng/ml) as previously described (29, 30). MMP-8, which is called neutrophil collagenase, is synthesized as a latent proenzyme during the myelocyte stage and stored within specific granules of neutrophils, ultimately being released from neutrophils during inflammation *in vivo* (29). AF culture results were present in 303 patients, and IAI results were available in 299 patients because IAIR was not examined in 10 patients due to the limited amount of remaining AF.

### Statistical Analysis

The Kruskal–Wallis test was used to compare the continuous variables, and Pearson's chi-square test was used to compare the categorical variables according to the progression of inflammation in CP. Moreover, linear by linear association was used to compare the frequency of inflammation in placental compartments (i.e., chorio-deciduitis, amnionitis, and funisitis) according to the progression of inflammation in CP. Spearman's rank correlation test was used to examine the correlation between IAIR and the progression of inflammation in CP. Pearson's chi-square test was used for the comparison of the frequency of IAI, positive AF culture, and suspected or proven EONS among four groups, and the linear by linear association test was used to investigate the trend. We performed multiple logistic regression analysis for the exploration of the relationship between various variables and EONS with the adjustment of potential confounders (i.e., GA at delivery, GA at amniocentesis, gender of newborn, preterm-PROM as a type of preterm delivery, and cesarean delivery) associated with inflammatory responses and parameters [i.e., parity (≥1), antenatal corticosteroid use, and antenatal antibiotics use] different among groups according to the progression of inflammation in CP. Statistical significance was defined as a *P* < 0.05.

## Results

### Clinical Characteristics and Pregnancy Outcomes According to the Progression of Inflammation Within the Chorionic Plate

Each stage (stage-0 to stage-3) in the progression of inflammation within CP was present in 69.3% (214/309), 15.9% (49/309), 11.0% (34/309), and 3.9% (12/309) of the study population, respectively (Table 1). We found a significant difference in GA at amniocentesis, GA at delivery and birth weight, and in the frequency of parity (≥1), 1-min Apgar score <7, 5-min Apgar score <7, antenatal corticosteroids use, and antenatal antibiotic use among stages according to the progression of inflammation in CP (Table 1). Of note, chorio-deciduitis, amnionitis, and funisitis gradually increased with increasing stage of inflammation in CP (Table 1). Moreover, we also found chorio-deciduitis, amnionitis, funisitis, suspected EONS, and proven EONS were significantly more frequent in cases with IAI than in those without IAI (Table 2).

**Table 1:** Clinical characteristics and pregnancy outcomes according to the progression of inflammation within the chorionic plate (CP).

	**Stage-0, inflammation-free CP**	**Stage-1, inflammation restricted to SCF**	**Stage-2, inflammation in the CT of CP without chorionic vasculitis**	**Stage-3, Chorionic vasculitis**	***P*^†^**
	**69.3% (*n =* 214)**	**15.9% (*n =* 49)**	**11.0% (*n =* 34)**	**3.9% (*n =* 12)**	
Maternal age, years (mean ± SD)	30.32 ± 4.45	29.24 ± 4.56	30.32 ± 4.45	31.00 ± 4.61	0.500
Parity (≥1)	56.5% (121/214)	40.8% (20/49)	55.9% (19/34)	83.3% (10/12)	0.044
GA at amniocentesis, weeks (mean ± SD)	34.05 ± 2.81	31.42 ± 3.32	29.46 ± 3.57	28.24 ± 4.57	<0.001
Types of preterm delivery					0.333
PTL	49.1% (105/214)	49.0% (24/49)	55.9% (19/34)	25.0% (3/12)	
Preterm-PROM	50.9% (109/214)	51.0% (25/49)	44.1% (15/34)	75.0% (9/12)	
GA at delivery, weeks (mean ± SD)	34.13 ± 2.81	31.55 ± 3.37	29.64 ± 3.51	28.41 ± 4.04	<0.001
Birth weight, g (mean ± SD)	2262.96 ± 604.13	1740.16 ± 630.12	1245.27 ± 600.07	1339.08 ± 675.43	<0.001
Male newborn	57.0% (122/214)	44.9% (22/49)	47.1% (16/34)	41.7% (5/12)	0.289
Apgar score at 1 min <7	29.4% (63/214)	42.9% (21/49)	76.5% (26/34)	83.3% (10/12)	<0.000001
Apgar score at 5 min <7	14.0% (30/214)	28.6% (14/49)	44.1% (15/34)	41.7% (5/12)	0.000058
Cesarean delivery	49.1% (105/214)	34.7% (17/49)	38.2% (13/34)	50.0% (6/12)	0.238
Funisitis^‡^	6.1% (13/214)	61.2% (30/49)	64.7% (22/34)	100% (12/12)	<0.000001
Amnionitis^‡^	4.7% (10/214)	46.9% (23/49)	76.5% (26/34)	83.3% (12/12)	<0.000001
Chorio-deciduitis^‡^	23.4% (50/214)	77.6% (38/49)	100% (34/34)	100% (12/12)	<0.000001
Antenatal corticosteroids use^∫^	25.7% (54/210)	38.8% (19/49)	35.5% (12/34)	58.3% (7/12)	0.034
Antenatal antibiotics use^∫∫^	50.2% (103/205)	66.0% (31/47)	72.7% (24/33)	72.7% (8/11)	0.022

† *Among the four groups, Kruskal–Wallis test was used for comparison of continuous variables, and Pearson's chi-square test was used for comparison of the proportions*.

‡ *Among the four groups, linear-by-linear association was used for comparison of the proportions*.

∫ *Of 309 cases, 305 patients were included in this analysis because the information about antenatal corticosteroids use in the medical records was omitted in four patients*.

∫∫ *Of 309 cases, 296 patients were included in this analysis because the information about antenatal antibiotics use in the medical records was omitted in 13 patients*.

*CP, chorionic plate; CT, connective tissue; GA, gestational age; preterm-PROM, preterm premature rupture of membranes; PTL, preterm labor and intact membranes; SCF, subchorionic fibrin*.

**Table 2:** Clinical characteristics, and pregnancy and neonatal outcomes according to the presence or absence of intra-amniotic inflammation (IAI).

	**Absence of IAI (*n =* 161)**	**Presence of IAI (*n =* 138)**	***P*^†^**
	**53.8% (161/299)^∮^**	**46.2% (*n =* 138/299)^∮^**	
Maternal age, years (mean ± SD)	30.18 ± 4.60	30.31 ± 4.30	0.721
Parity (≥1)	55.3% (89/161)	55.8% (77/138)	1.000
GA at amniocentesis, weeks (mean ± SD)	34.41 ± 2.40	31.21 ± 3.94	<0.001
Causes of preterm delivery			0.064
PTL	43.5% (70/161)	54.3% (75/138)	
Preterm-PROM	56.5% (91/161)	45.7% (63/138)	
GA at delivery, weeks (mean ± SD)	34.50 ± 2.39	31.33 ± 3.92	<0.001
Birth weight, g (mean ± SD)	2358.27 ± 572.08	1727.00 ± 667.48	<0.001
Male newborn	60.2% (97/161)	46.4% (64/138)	0.020
Apgar score at 1 min <7	25.5% (41/161)	52.9% (73/138)	<0.000005
Apgar score at 5 min <7	11.2% (18/161)	32.6% (45/138)	0.000008
Cesarean delivery	50.9% (82/161)	38.4% (53/138)	0.036
Funisitis	5.0% (8/161)	45.7% (63/138)	<0.000001
Amnionitis	1.9% (3/161)	44.2% (61/138)	<0.000001
Chorio-deciduitis	16.1% (26/161)	73.2% (101/138)	<0.000001
Antenatal corticosteroids use^∫^	23.9% (38/159)	37.5% (51/136)	0.015
Antenatal antibiotics use^∫∫^	51.6% (80/155)	61.1% (80/131)	0.121
Positive AF culture^‡^	6.9% (11/160)	31.6% (42/133)	<0.000001
Suspected EONS^††^	4.6% (7/152)	18.3% (23/126)	0.000342
Proven EONS^††^	0% (0/152)	6.3% (8/126)	0.002
Suspected or proven EONS^††^	4.6% (7/152)	18.3% (23/126)	0.000342

∮ *IAI results were available in 299 patients, and those were not examined in 10 patients due to the limited amount of remaining AF*.

† *Between two groups, the Mann–Whitney U-test was used for comparison of continuous variables, and Fisher's exact test was used for comparison of the proportions*.

∫ *Of 299 cases, 295 patients were included in this analysis, because the information about antenatal corticosteroids use in the medical records was omitted in four patients*.

∫∫ *Of 299 cases, 286 patients were included in this analysis because the information about antenatal antibiotics use in the medical records was omitted in 13 patients*.

‡ *Of 299 cases, AF culture results were available in 293 patients*.

†† *Twenty-one neonates were excluded from the analysis of EONS among 299 cases because they died shortly after delivery as a result of extreme prematurity (n = 11) or had no information about EONS in the medical records (n = 10) and thus could not be evaluated with respect to the presence or absence of EONS*.

*AF, amniotic fluid; CP, chorionic plate; CT, connective tissue; EONS, early-onset neonatal sepsis; GA, gestational age; IAI, intra-amniotic inflammation; preterm-PROM, preterm premature rupture of membranes; PTL, preterm labor and intact membranes; SCF, subchorionic fibrin*.

### Amniotic Fluid Matrix Metalloproteinase-8 Concentrations, and the Frequency of Intra-Amniotic Inflammation and Positive Amniotic Fluid Culture According to the Progression of Inflammation Within the Chorionic Plate

**Figure 1:**
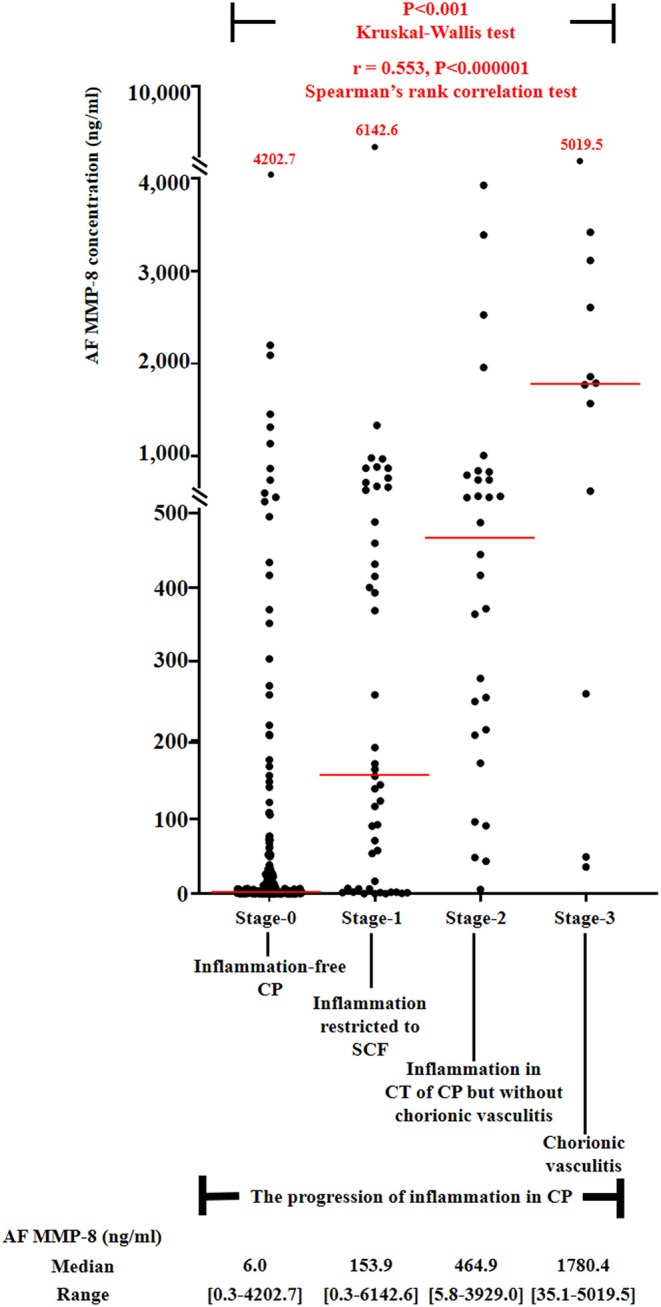
Amniotic fluid (AF) MMP-8 concentrations according to the progression of inflammation within chorionic plate (CP) [stage-0, inflammation-free CP vs. stage-1, inflammation restricted to subchorionic fibrin (SCF) vs. stage-2, inflammation in the connective tissue (CT) of CP without chorionic vasculitis vs. stage-3, chorionic vasculitis; median, range, 6.0 ng/ml (0.3–4202.7 ng/ml) vs. 153.9 ng/ml (0.3–6142.6 ng/ml) vs. 464.9 ng/ml (5.8–3929.0 ng/ml) vs. 1780.4 ng/ml (35.1–5019.5 ng/ml)] among preterm gestations. Each *P*-value is shown in the graph. AF MMP-8 concentration results were available in 299 patients, and those were not examined in 10 patients due to the limited amount of remaining AF.

**Figure 2:**
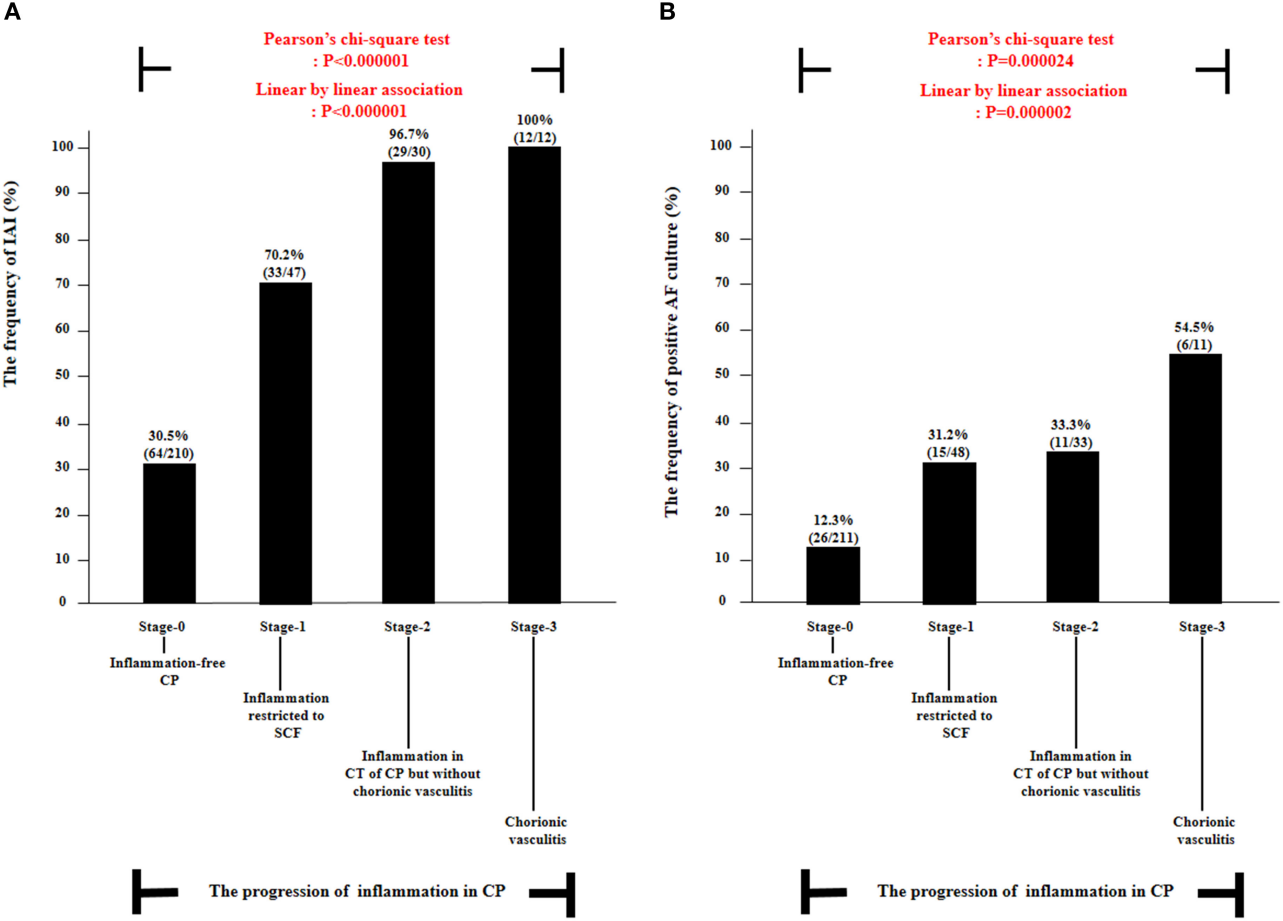
The frequency of intra-amniotic inflammation (IAI) **(A)** and positive amniotic fluid (AF) culture **(B)** according to the progression of inflammation within chorionic plate (CP) [i.e., stage-0, inflammation-free CP vs. stage-1, inflammation restricted to subchorionic fibrin (SCF) vs. stage-2, inflammation in the connective tissue (CT) of CP without chorionic vasculitis vs. stage-3, chorionic vasculitis] among preterm gestations. Each *P*-value is shown in the graph. AF culture results were available in 303 patients. IAI results were available in 299 patients, and those were not examined in 10 patients due to the limited amount of AF remaining.

Figure 1 demonstrated the correlation between AF MMP-8 levels and the progression of inflammation in CP. AF MMP-8 levels gradually elevated according to the progression of inflammation in CP (Kruskal–Wallis test, *P* < 0.001; Spearman's rank correlation test, *P* < 0.000001, γ = 0.553). Moreover, there was a significantly stepwise elevation in the frequency of IAI (Figure 2A) and positive AF culture (Figure 2B) according to the progression of inflammation in CP (each for *P* < 0.00005 in Pearson's chi-square test and *P* < 0.000005 in a linear-by-linear association). Table 3 demonstrated the types of microorganisms that were isolated from AF according to the progression of inflammation in CP. Genital mycoplasmas (i.e., Ureaplasmas and Mycoplasmas) were the most common isolates (65.5%; 38/58) in positive AF culture (Table 3). It is interesting that genital mycoplasma was not found in any cases with chorionic vasculitis (stage-3) (Table 3).

**Table 3:** Types of microorganisms isolated from the amniotic fluid (AF) according to the progression of inflammation in the chorionic plate (CP)^a^.

**The progression of inflammation in CP**	**Stage-0 inflammation-free CP**	**Stage-1 inflammation restricted to CP**	**Stage-2 inflammation in CT of CP without chorionic vasculitis**	**Stage-3 Chorioic vasculitis**
	***n =* 211**	***n =* 48**	***n =* 33**	***n =* 11**
Types of micro-organisms	Numbers of positive AF culture in each stage-group			
	*n =* 26	*n =* 15	*n =* 11	*n =* 6
1. Genital mycoplasmas	17	10	7	0
Ureaplasmas	16	9	7	0
*M. hominis*	1	0	0	0
Ureaplasmas and *M. hominis*	0	1	0	0
2. Genital mycoplasmas with other microorganisms	3	2	1	0
Ureaplasmas and *Staphylococcus* species	1	1	0	0
Ureaplasmas and *Streptococcus* species	1	1	0	0
Ureaplasmas, *Streptococcus* species, and *Candida* species	0	0	1	0
*M. hominis* and *E. coli*	1	0	0	0
3. Other microorganisms	6	3	3	6
*Lactobacillus* species	3	0	0	0
*Staphylococcus* species	0	1	1	0
*Streptococcus* species	0	1	1	1
*Corynebacterium*	1	0	0	0
*Candida* species	1	1	0	2
*E. faecalis* and *P. aeruginosa*	1	0	0	0
*E. coli*	0	0	0	1
*A. baumani, E. coli*, and *Staphylococcus* species	0	0	1	0
*Turolopsis glabrata*	0	0	0	1
Not available	0	0	0	1

a *AF culture results were available in 303 patients*.

### Early-Onset Neonatal Sepsis According to the Progression of Inflammation Within Chorionic Plate

**Figure 3:**
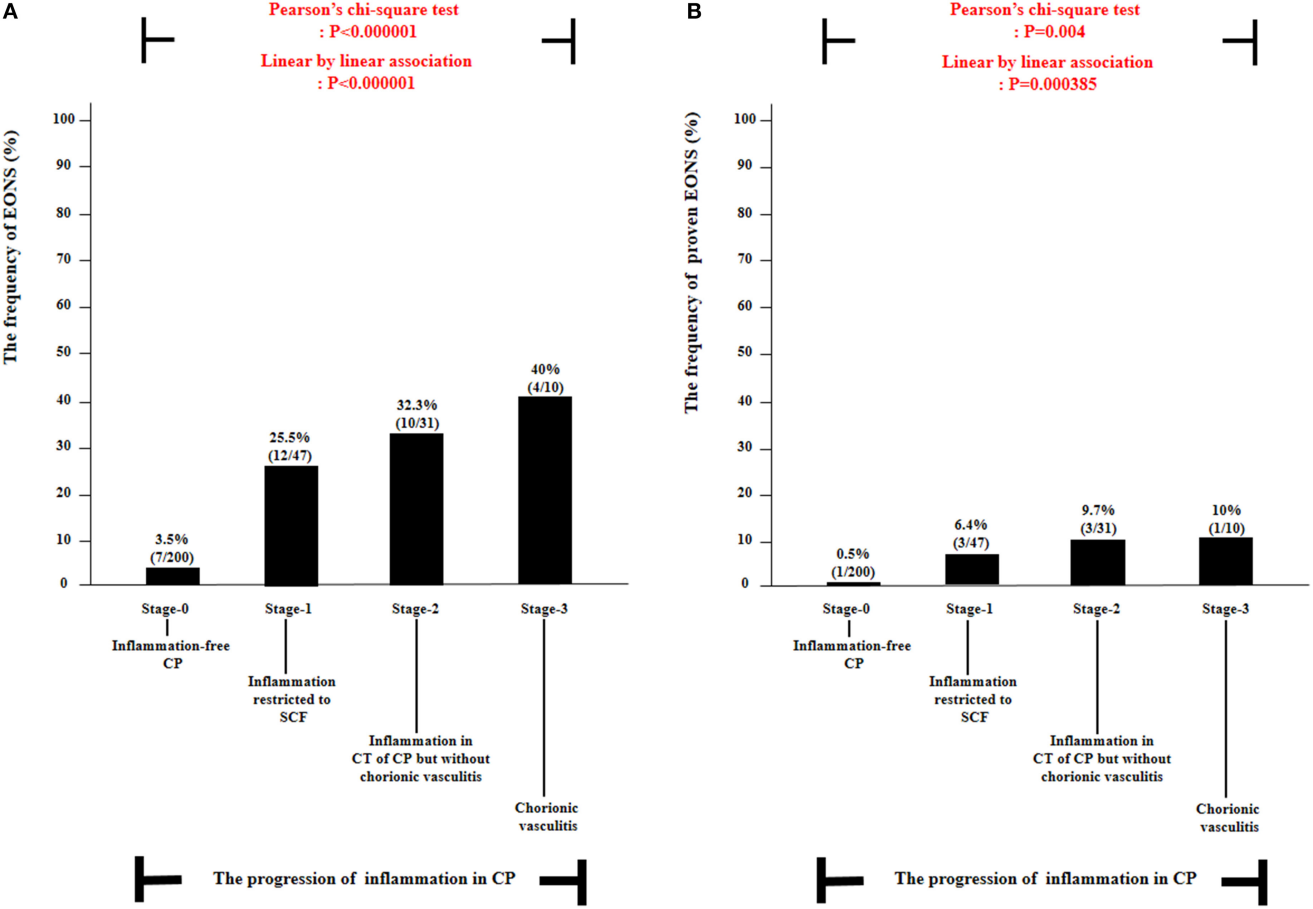
The frequency of suspected or proven early-onset neonatal sepsis (EONS) **(A)** and proven EONS **(B)** according to the progression of inflammation within chorionic plate (CP) [i.e., stage-0, inflammation-free CP vs. stage-1, inflammation restricted to subchorionic fibrin (SCF) vs. stage-2, inflammation in the connective tissue (CT) of CP without chorionic vasculitis vs. stage-3, chorionic vasculitis] among preterm gestations. Each *P*-value is shown in the graph. Twenty-one neonates were excluded from the analysis of suspected or proven EONS among 309 cases because they died shortly after delivery as a result of extremely prematurity (*n* = 11) or had no information about EONS in the medical records (*n* = 10) and thus could not be evaluated with respect to the presence or absence of EONS.

Table 4 describes the clinical characteristics and pregnancy outcomes according to the presence or absence of suspected or proven EONS. There was a significant difference in GA at amniocentesis, GA at delivery and birth weight, and in the frequency of 1-min Apgar score <7, 5-min Apgar score <7, and inflammation in each placental compartment (i.e., chorio-deciduitis, amnionitis, funisitis, and inflammation in CP). Figure 3 illustrated the relationship between the progression of inflammation in CP and the frequency of suspected or proven EONS (Figure 3A) and proven EONS (Figure 3B). The frequency of suspected or proven EONS (Figure 3A) and proven EONS (Figure 3B) continuously increased with the progression of inflammation in CP (each for *P* < 0.005 in Pearson's chi-square test and each for *P* < 0.0005 in a linear-by-linear association). Moreover, Supplementary Figure 2 shows a significant step-wise increase in the frequency of suspected or proven EONS, or immediate neonatal death shortly after birth (Supplementary Figure 2A), proven EONS or immediate neonatal death shortly after birth (Supplementary Figure 2B), and immediate neonatal death shortly after birth (Supplementary Figure 2C) according to the progression of inflammation within CP (each for *P* < 0.05 in a linear-by-linear association). Supplementary Figure 3 shows the predicted probability of suspected or proven EONS, or immediate neonatal death shortly after delivery according to GA at delivery and the presence or absence of inflammation in CP among 302 cases with GA at delivery (≥24 weeks) of the study population. Logistic regression analysis demonstrated that the presence of inflammation in CP was related to an increase in suspected or proven EONS, or immediate neonatal death shortly after delivery even after adjusting for GA at delivery [odds ratio (OR) = 5.887, 95% confidence interval (CI) 2.448–14.155, *P* = 0.000075; Supplementary Figure 3]. Notably, multiple logistic regression analysis demonstrated that each stage in the progression of inflammation within CP was a risk factor for the development of suspected or proven EONS compared with stage-0 even after the adjustment for potential confounding variables such as GA at delivery and GA at amniocentesis, and moreover, a more advanced stage in the progression of inflammation within CP was associated with a higher OR for the development of suspected or proven EONS in univariate analysis (Table 5) and multiple logistic regression analysis [Table 6; stage-1 vs. stage-2 vs. stage-3; OR, 7.976, 95% CI (2.296–27.714) vs. OR, 9.936, 95% CI (2.361–41.814) vs. OR, 34.916, 95% CI (2.701–451.394)].

**Table 4:** Clinical characteristics and pregnancy outcomes according to the presence or absence of suspected or proven early-onset neonatal sepsis (EONS)^a^.

	**Suspected or proven EONS (−) (*n =* 255) 88.5% (255/288)**	**Suspected or proven EONS (+) (*n =* 33)11.5% (33/288)**	***P*-value**
Maternal age, years (mean ± SD)	30.10 ± 4.52	29.85 ± 4.14	0.840
Parity (≥1)	54.5% (139/255)	54.5% (18/33)	1.000
GA at amniocentesis, weeks (mean ± SD)	33.49 ± 2.89	30.92 ± 3.50	<0.001
Causes of preterm delivery			0.713
PTL	47.5% (121/255)	51.5% (17/33)	
Preterm-PROM	52.5% (134/255)	48.5% (16/33)	
GA at delivery, weeks (mean ± SD)	33.59 ± 2.89	31.09 ± 3.39	<0.001
Birth weight, g (mean ± SD)	2148.50 ± 599.28	1672.19 ± 623.81	<0.001
Male newborn	54.5% (139/255)	45.5% (15/33)	0.358
1-min Apgar score of <7	32.5% (83/255)	69.7% (23/33)	0.000071
5-min Apgar score of <7	14.1% (36/255)	48.5% (16/33)	0.000017
Cesarean delivery	45.5% (116/255)	45.5% (15/33)	1.000
Funisitis	19.6% (50/255)	69.7% (23/33)	<0.000001
Amnionitis	16.1% (41/255)	69.7% (23/33)	<0.000001
Chorio-deciduitis	38.0% (97/255)	84.8% (28/33)	<0.000001
Inflammation in CP	10.6% (27/255)	42.4% (14/33)	0.000019
Antenatal corticosteroids use^b^	30.7% (77/251)	36.4% (12/33)	0.551
Antenatal antibiotics use^c^	56.3% (138/245)	58.1% (18/31)	1.000

*CP, chorionic plate; EONS, early-onset neonatal sepsis; GA, gestational age; preterm-PROM, preterm premature rupture of membranes; PTL, preterm labor and intact membranes; SD, standard deviation*.

a *Twenty-one neonates were excluded from the analysis of EONS among 309 cases because they died shortly after delivery as a result of extremely prematurity (n = 11) or had no information about EONS in the medical records (n = 10) and thus could not be evaluated with respect to the presence or absence of EONS*.

b *Of 288 cases, 284 patients were included in this analysis because the information about antenatal corticosteroids use in the medical records was omitted in four patients*.

c *Of 288 cases, 276 patients were included in this analysis because the information about antenatal antibiotics use in the medical records was omitted in 12 patients*.

**Table 5:** The relationship between a stage-group according to the progression of inflammation in the chorionic plate (CP) and suspected or proven early-onset neonatal sepsis (EONS) when each stage-group was compared with stage-0 group (inflammation-free CP) by univariate analysis.

		**OR**	**95% CI**	***P*-value**
Presence or absence of inflammation in CP and the stage according to the progression of inflammation in CP.	Stage-1, inflammation restricted to SCF	9.453	(3.480, 25.678)	0.000011
	Stage-2, inflammation in CT of CP	13.129	(4.523, 38.113)	0.000002
	Stage-3, chorionic vasculitis	18.381	(4.214, 80.171)	0.000107

*EONS, early-onset neonatal sepsis; CP, chorionic plate; CT, connective tissue; SCF, subchorionic fibrin*.

**Table 6:** The relationship between various variables and suspected or proven early-onset neonatal sepsis (EONS) by multiple logistic regression analysis among groups according to the progression of inflammation in the chorionic plate (CP).

		**OR**	**95% CI**	***P*-value**
Stage-1, inflammation restricted to SCF vs. Stage-0, inflammation-free CP	Stage-1, inflammation restricted to SCF	7.976	(2.296, 27.714)	0.001
	GA at delivery	0.124	(0.001, 29.492)	0.454
	GA at amniocentesis	6.639	(0.027, 1650.059)	0.501
	Antenatal corticosteroids use	1.229	(0.339, 4.457)	0.753
	Antenatal antibiotics use	0.389	(0.093, 1.624)	0.195
	Preterm-PROM as a type of preterm delivery	1.773	(0.420, 7.487)	0.436
	Parity (≥1)	0.803	(0.248, 2.605)	0.715
	Male gender of newborn	0.647	(0.209, 2.002)	0.450
	Cesarean delivery	1.303	(0.406, 4.182)	0.656
Stage-2, inflammation in CT of CP but without chorionic vasculitis vs. Stage-0, inflammation-free CP	Stage-2, inflammation in CT of CP but without chorionic vasculitis	9.936	(2.361, 41.814)	0.002
	GA at delivery	2.663	(0.330, 21.494)	0.358
	GA at amniocentesis	0.328	(0.041, 2.593)	0.291
	Antenatal corticosteroids use	1.240	(0.328, 4.688)	0.752
	Antenatal antibiotics use	0.417	(0.087, 1.993)	0.273
	Preterm-PROM as a type of preterm delivery	1.097	(0.242, 4.980)	0.905
	Parity (≥1)	0.542	(0.159, 1.847)	0.327
	Male gender of newborn	0.736	(0.226, 2.396)	0.611
	Cesarean delivery	0.665	(0.188, 2.353)	0.527
Stage-3, chorionic vasculitis vs. Stage-0, inflammation-free CP	Stage-3, chorionic vasculitis	34.916	(2.701, 451.394)	0.007
	GA at delivery	0.115	(0.000, 157.457)	0.557
	GA at amniocentesis	8.183	(0.006, 11881.509)	0.571
	Antenatal corticosteroids use	1.655	(0.328, 8.352)	0.542
	Antenatal antibiotics use	0.278	(0.026, 2.942)	0.288
	Preterm-PROM as a type of preterm delivery	0.423	(0.040, 4.427)	0.472
	Parity (≥1)	0.986	(0.196, 4.960)	0.986
	Male gender of newborn	0.293	(0.059, 1.448)	0.132
	Cesarean delivery	0.523	(0.097, 2.816)	0.451

*EONS, early-onset neonatal sepsis; CP, chorionic plate; CT, connective tissue; SCF, subchorionic fibrin; GA, gestational age; preterm-PROM, preterm premature rupture of membranes*.

### The Progression of Inflammation Within Chorionic Plate

Supplementary Figure 4 shows inflammation-free CP (Supplementary Figure 4A), inflammation restricted to SCF (Supplementary Figure 4B), inflammation in the CT of CP without chorionic vasculitis (Supplementary Figure 4C), and chorionic vasculitis (Supplementary Figure 4C) in H & E-stained histologic sections of CP.

## Discussion

### Principal Findings

The principal findings of this study were that IAIR is more likely and severe according to the progression of inflammation in CP (i.e., inflammation-free CP, inflammation restricted to SCF, inflammation in the CT of CP without chorionic vasculitis, and chorionic vasculitis), and this progression of inflammation in CP is an independent risk factor for the development of EONS in neonates delivered due to either PTL or preterm-PROM.

### Limitations in Previous Classifications of Acute Inflammation in the Chorionic Plate

There are various classifications of acute inflammation in CP as an independent compartment (Supplementary Table 1). However, all had limitations as follows. First, most studies did not include inflammation restricted to the SCF as an initial stage of inflammation in CP (13, 15, 16, 18–23). Instead, some studies included inflammation in IVS (i.e., subchorionic IVS, or between decidua and CP) as an early stage of inflammation in CP (13, 15, 16, 18, 19, 21), although IVS is regarded as placenta parenchyma but not CP. Second, most studies did not include the inflammation in the CT of CP (18, 21, 23) or chorionic vasculitis (13, 15, 16, 18, 19, 21–23), and did not differentiate between inflammation in the CT of CP and chorionic vasculitis (14, 17).

### No Previous Study Demonstrated the Correlation Between Intra-Amniotic Inflammatory Response and the Progression of Inflammation Within the Full-Detailed Subdivisions of the Chorionic Plate

Several previous studies reported that IAIR gradually elevated according to the progression of inflammation in CP (Supplementary Table 1) (13, 15, 16, 18, 19, 21, 22). However, all those studies did not include both the inflammation restricted to SCF as an early stage inflammation and chorionic vasculitis as an advanced stage inflammation. Therefore, those studies could not provide the exact intensity of IAIR with the progression of inflammation within the full-detailed subdivisions of CP as an independent compartment. Of note, up to now, there are only three classifications with the full-detailed subdivisions in CP (28, 31, 32). Inflammation in CP was divided into maternal inflammatory response (MIR) (i.e., stage 1, inflammation in SCF and stage 2, inflammation in fibrous chorion) and fetal inflammatory response (FIR) (i.e., stage 1, chorionic vasculitis) in the “Amsterdam staging system” (31), and Blanc (32) and Salafia et al. (28) included the full-detailed compartments for the analysis of inflammation in CP (i.e., Blanc WA: subchorionic IVS, placental chorion, CVs, and amnion; and Salafia et al. inflammation-free CP, mild inflammation-restricted to SCF, severe inflammation-restricted to SCF, inflammation in the CT of CP without chorionic vasculitis, and chorionic vasculitis). Unfortunately, those studies did not examine IAIR. Moreover, there was no study reporting a stepwise elevation in positive AF culture according to the progression of inflammation within the full-detailed subdivisions of CP. To my knowledge, we first demonstrated that IAIR gradually increased with the progression of inflammation in the full-detailed subdivisions of CP.

### Progression of Inflammation Within the Chorionic Plate May Be a Reliable Indicator for the Severity of Intra-Amniotic Inflammatory Response

Maternal neutrophils that escaped from maternal decidual vessels invade extraplacental membranes (i.e., decidua parietalis, chorion, and amnion), and fetal neutrophils from umbilical vein and/or arteries infiltrate into umbilical vessel walls and Wharton's jelly of umbilical cord during ascending intrauterine infection (33). Moreover, maternal neutrophils *via* IVS and fetal neutrophils through CVs can migrate to the CT in CP featuring the amniotropism by ascending intrauterine infection (34). Indeed, both fetal and maternal neutrophils are shown in the progression of inflammation in CP (17), while pure fetal and maternal neutrophils are exclusively found in umbilical cord and extraplacental membranes, respectively, in the context of ascending intrauterine infection (35, 36). Given that acute inflammation simultaneously develops in each placental compartment (i.e., extraplacental membranes, umbilical cord, and CP) during the progression of ascending intrauterine infection, AF neutrophils in the context of IAI is likely to show the mixture of fetal and maternal neutrophils migrated from each placental compartment. Indeed, AF neutrophils are known to be either predominantly of fetal or maternal origin, in women with IA infection and/or inflammation (37). Therefore, considering the mixed pattern of fetal and maternal neutrophils in both CP and AF during ascending intrauterine infection, the progression of inflammation within CP as an independent compartment for the analysis of acute inflammation may be a reliable indicator for the severity of IAIR.

### A More Advanced Stage in the Progression of Inflammation in the Chorionic Plate Is Associated With a Higher Odds Ratio for the Development of Early-Onset Neonatal Sepsis Even After the Adjustment for Potential Confounding Variables Such as Gestational Age at Delivery

We did not find any previous studies about a stepwise increase in the frequency of EONS according to the progression of inflammation in the full-detailed subdivisions of CP even after the adjustment for potential confounding variables such as GA at delivery (Supplementary Table 1). However, this finding in the current study may indicate that CP can be an independent compartment for the assessment of infectious neonatal morbidities (i.e., EONS), although CP continues to be denigrated compared with either the extraplacental membranes or umbilical cord.

### Inflammation-Free Chorionic Plate Cannot Reflect Inflammation-Free Extraplacental Membranes

Notably, chorio-deciduitis was found in about one-fourth of the cases with inflammation-free CP [23.4% (50/214)] suggesting that inflammation-free CP cannot reflect inflammation-free extraplacental membranes. This finding is easily explained by the more adjacent location of the chorio-decidua in extraplacental membranes than CP to the cervical canal leading to the conclusion that acute inflammation during ascending intrauterine infection is more likely to develop in the chorio-decidua than in the CP. Indeed, the frequency of chorio-deciduitis was decreased considerably to 6.3% (1/16) in patients with inflammation-free CP in the context of placenta previa among the current study population (data are not shown). In other words, inflammation-free extraplacental membranes were found in more than 90% [93.8% (15/16)] of the cases with inflammation-free CP in the context of placenta previa (data are not shown). Given that CP is directly exposed to the cervical canal, and extraplacental membranes are more remote from the cervical canal than CP in the placenta previa, one can expect that the inflammation-free CP may reflect inflammation-free extraplacental membranes in cases with placenta previa.

### Strengths and Weaknesses of the Study

This study had major strengths. First, it included a large cohort of singleton preterm gestations who had results of AF study and placental histology with full-detailed subdivisions of CP (*n* = 309). Although a previous study examined the relationship between IAIR and progression of inflammation in CP in a large study population (*n* = 428) (22), they included term births in addition to preterm births and did not classify CP into the full-detailed subdivisions. Second, this study reaffirmed a clear correlation between IAIR and the progression of inflammation in CP by showing a stepwise increase in the frequency of several surrogate markers for ascending intrauterine infection (i.e., positive AF culture, choriodeciduitis, amnionitis, and funisitis) according to the progression of inflammation in CP. The weaknesses of the current study are as follows. First, we included the results of placenta histologic examination and AF in 309 singleton pregnant women with spontaneous preterm birth from February 1993 to April 2004, and therefore, we should explain the stability about the documentation of placental histopathologic reports and the measurement of AF MMP-8. However, the placental histopathologic reports have been routinely documented with the same diagnostic criteria (14, 28) within 1 week after delivery in all pregnant women-delivered preterm neonates in our institute. Moreover, we already finished the measurement of AF MMP-8 concentration in most study populations before the year 2006. Of note, at the year 2000, we first measured MMP-8 in AF obtained and stored in polyprophylene tubes at 70°C from most patients enrolled into our biobank between January 1993 and December 1999 for our previous two studies (29, 38), and verified that there was no significant difference in the median concentration of AF MMP-8 among the sampling year in the same context of situation (i.e., asymptomatic mid-trimester pregnant women). Furthermore, since 2000, we usually finished the measurement of MMP-8 in AF stored in polyprophylene tubes at 70°C within 1 year after the initial enrollment into our biobank for our previous studies about IAIR gauged by AF MMP-8 concentration in the context of singleton preterm birth. Second, we did not demonstrate a significant difference in the frequency of several surrogate markers for ascending intrauterine infection (i.e., IAI, positive AF culture, and EONS) between stage-1 and stage-2, and between stage-1 and stage-3, and a significant linear trend among stages-1 to -3 after removing the no inflammation group (stage-0, inflammation-free CP) according to the progression of inflammation in the full-detailed subdivisions of CP due to a small sample size for some groups (i.e., stage-2 and stage-3). However, the scope of our current study is to examine whether “there is a stepwise increase in several surrogate markers for ascending intrauterine infection (i.e., IAI, positive AF culture, and EONS),” but not “there is a significant difference in several surrogate markers for ascending intrauterine infection between each group,” according to the progression of inflammation in the full-detailed subdivisions of CP. Moreover, we must include at least 1,000–3,000 cases as study population to show a significant difference in several surrogate markers for ascending intrauterine infection between each stage group and a significant linear trend among stages-1 to -3 after removing the no inflammation group (stage-0, inflammation-free CP) according to the progression of inflammation in the full-detailed subdivisions of CP. Unfortunately, although only the national registry (big data) can include this tremendous number of study population, it cannot have something like our current data (i.e., AF and placenta histologic examination results).

### Clinical Implication

This is the first report showing that there is a stepwise increase in IAIR and several surrogate markers for ascending intrauterine infection [i.e., positive AF culture, inflammation in each placental compartment (i.e., choriodeciduitis, amnionitis, and funisitis) and EONS] according to the progression of inflammation in the full-detailed subdivisions of CP as an independent compartment for the assessment of acute inflammation. This finding suggests that CP may be another playground for the progression of ascending intrauterine infection in addition to extraplacental membranes, and the assessment for the progression of inflammation in CP may be a method for the prediction of EONS in spontaneous PTBs.

### Unanswered Question and Further Study

Either chorionitis (inflammation in membranous trophoblast of chorion within extraplacental membranes) or subchorionitis (inflammation in SCF within CP) is classified as MIR stage 1 in the Amsterdam Staging System, an early stage of acute placental inflammation (31). Indeed, our previous report demonstrated that IAIR was more intense in cases with inflammation restricted to chorio–decidua than in those with inflammation-free extraplacental membranes within extraplacental membranes (11), and the current study shows that IAIR was more severe in patients with inflammation restricted to SCF than in those with inflammation-free CP within CP. However, there is no information on which is associated with a more intense IAIR and a higher frequency of surrogate markers for ascending intrauterine infection (i.e., positive AF culture and funisitis) between inflammation restricted to chorio–decidua within extraplacental membranes or inflammation restricted to SCF within CP among preterm gestations with acute placental inflammation. The investigation to answer that question is likely to be a trigger for the exploration of targets for early intervention in patients at risk for spontaneous PTB.

## Data Availability Statement

All datasets generated/analyzed for this study are included in the manuscript.

## Ethics Statement

The studies involving human participants were reviewed and approved by the Institutional Review Board of our institute (SNUH). The patients/participants provided their written informed consent to participate in this study.

## Author Contributions

C-WP is the guarantor of the integrity of the entire study. C-WP, J-WO, and KM performed the literature research as well as the statistical analysis. All authors contributed to the study concepts and design, performed data acquisition or data analysis/interpretation, drafted, edited, and revised the manuscript for important intellectual content, approved the final version of the submitted manuscript, and agreed to be accountable for all aspects of the work in ensuring that questions related to the accuracy or integrity of any part of the work are appropriately investigated and resolved.

## Conflict of Interest

The authors declare that the research was conducted in the absence of any commercial or financial relationships that could be construed as a potential conflict of interest.

## Abbreviations

AF: amniotic fluid
CP: chorionic plate
CT: connective tissue
CVs: chorionic vessels
EONS: early-onset neonatal sepsis
FIR: fetal inflammatory response
GA: gestational age
IAI: intra-amniotic inflammation
IAIR: intra-amniotic inflammatory response
IVS: inter-villous space
MIR: maternal inflammatory response
MMP-8: matrix metalloproteinase-8
Preterm-PROM: preterm premature rupture of membranes
PTL: preterm labor and intact membranes
SCF: sub-chorionic fibrin.
