# Supplementary figures and images for: The Relationship Among Intra-Amniotic Inflammatory Response, The Progression of Inflammation in Chorionic Plate and Early-Onset Neonatal Sepsis
Source: Front Pediatr. 2021 Apr 29;9:582472. doi: 10.3389/fped.2021.582472 (PMC8116513; doi:10.3389/fped.2021.582472)

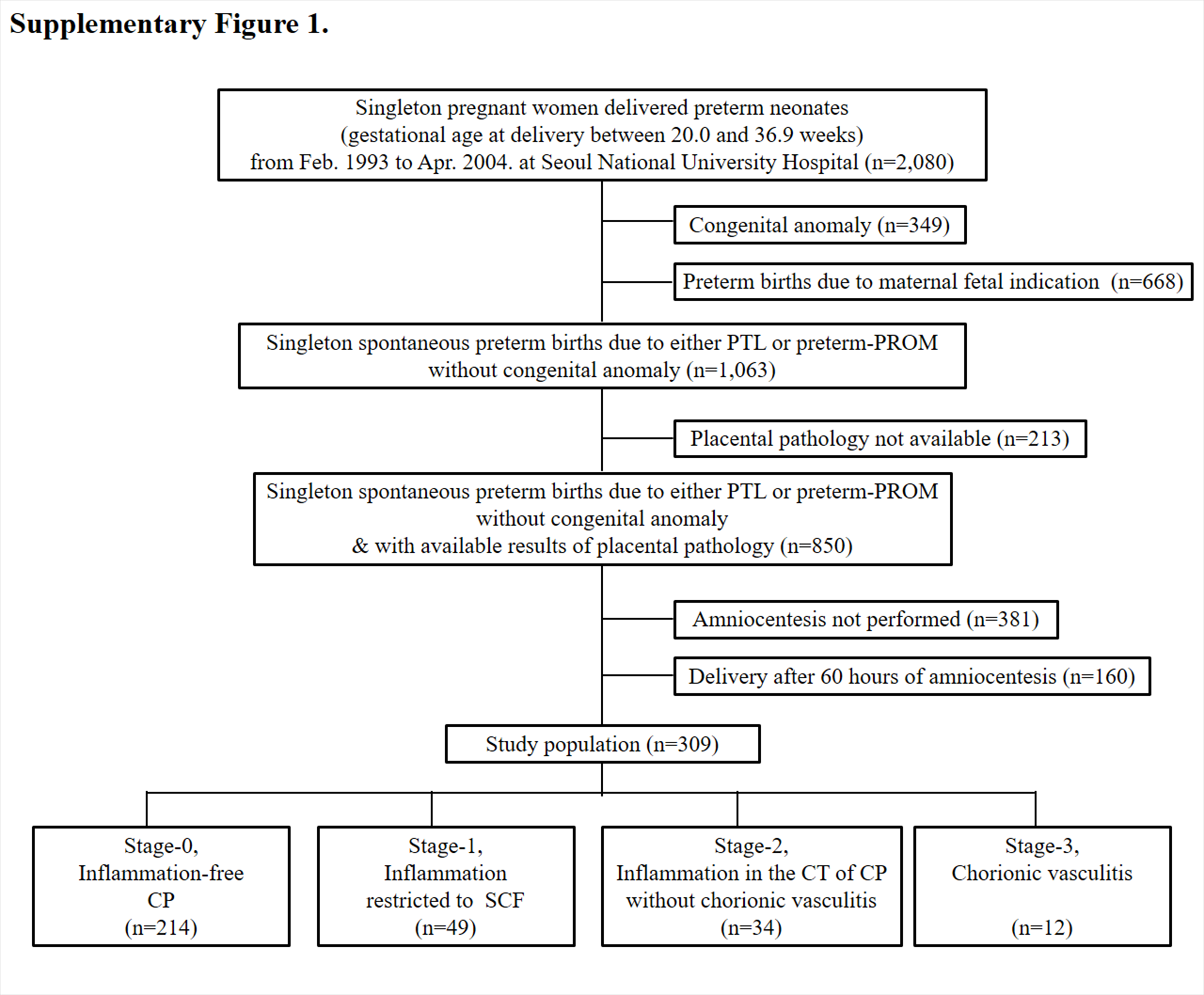

Supplement: Supplementary file 1 [file Image_1.TIF]

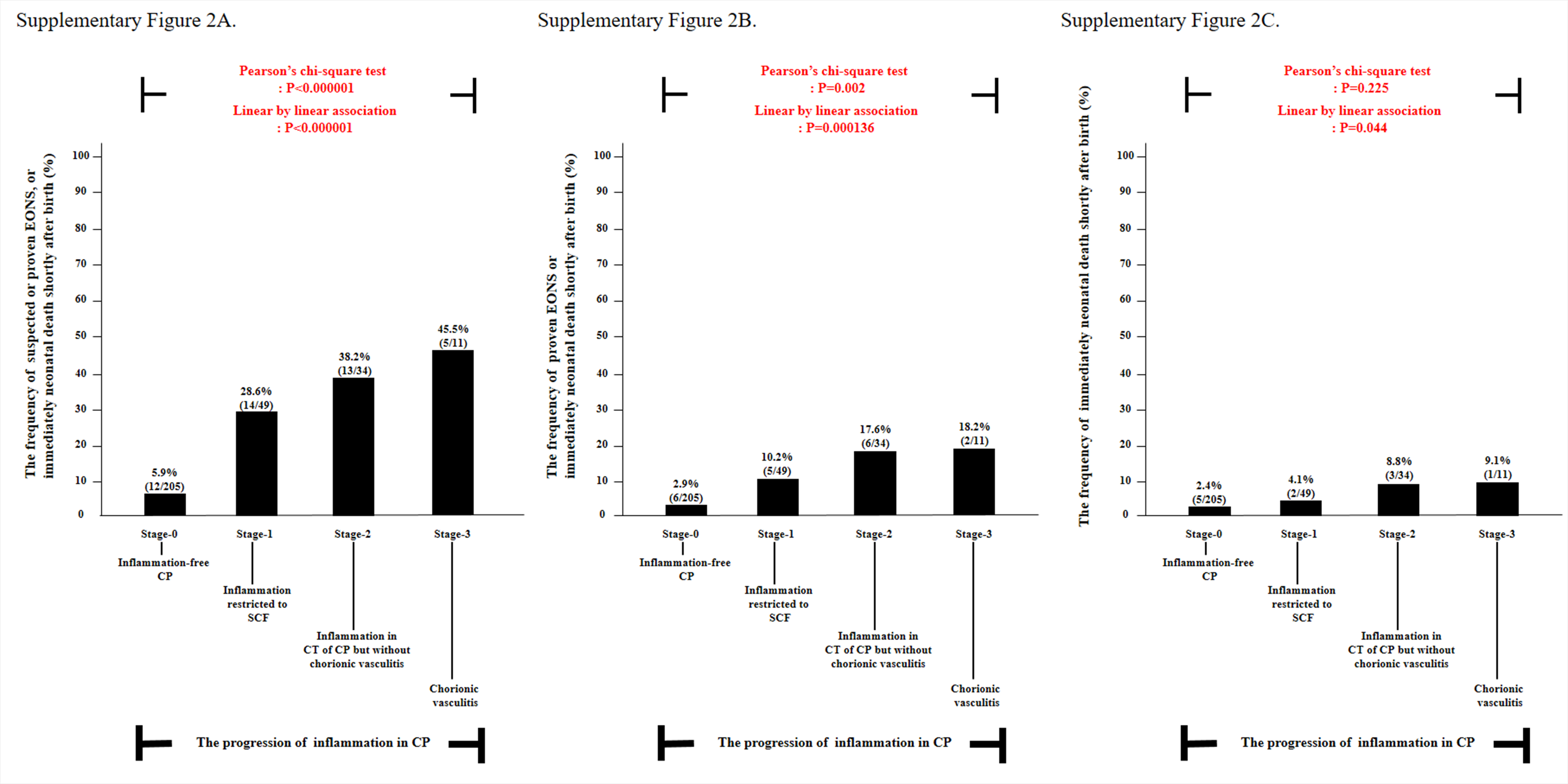

Supplement: Supplementary file 2 [file Image_2.TIF]

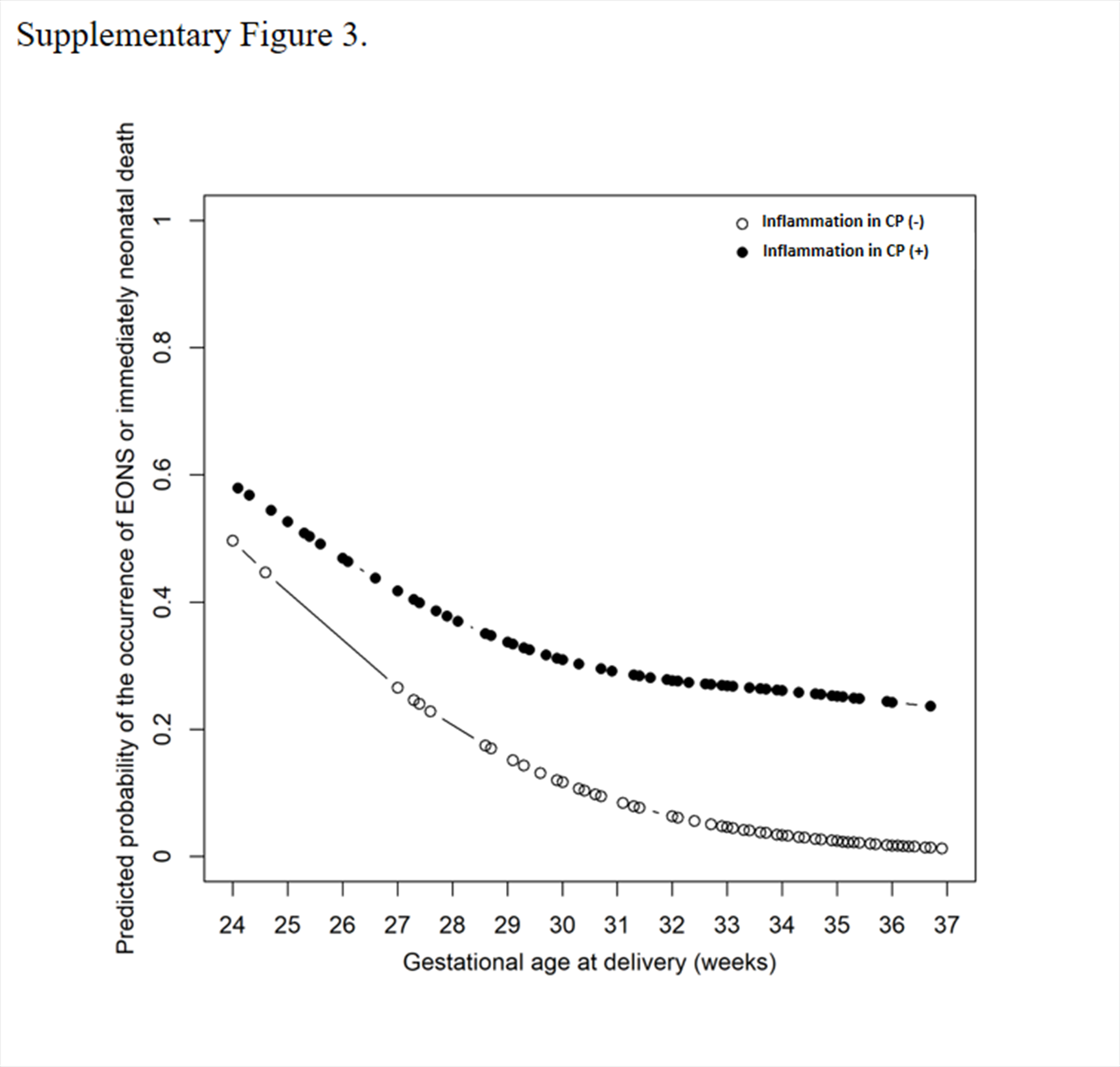

Supplement: Supplementary file 3 [file Image_3.TIF]

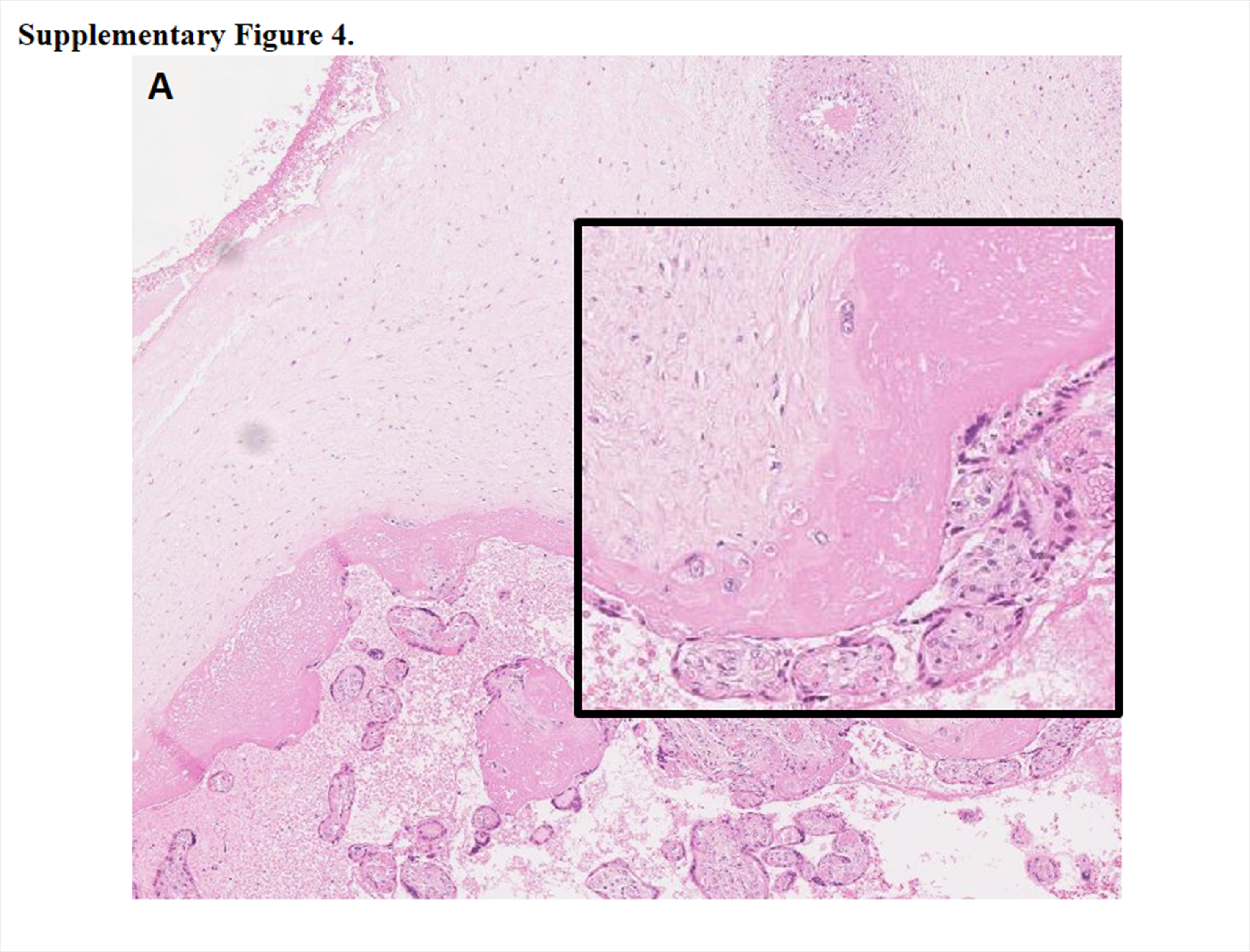

Supplement: Supplementary file 4 [file Image_4.TIF]

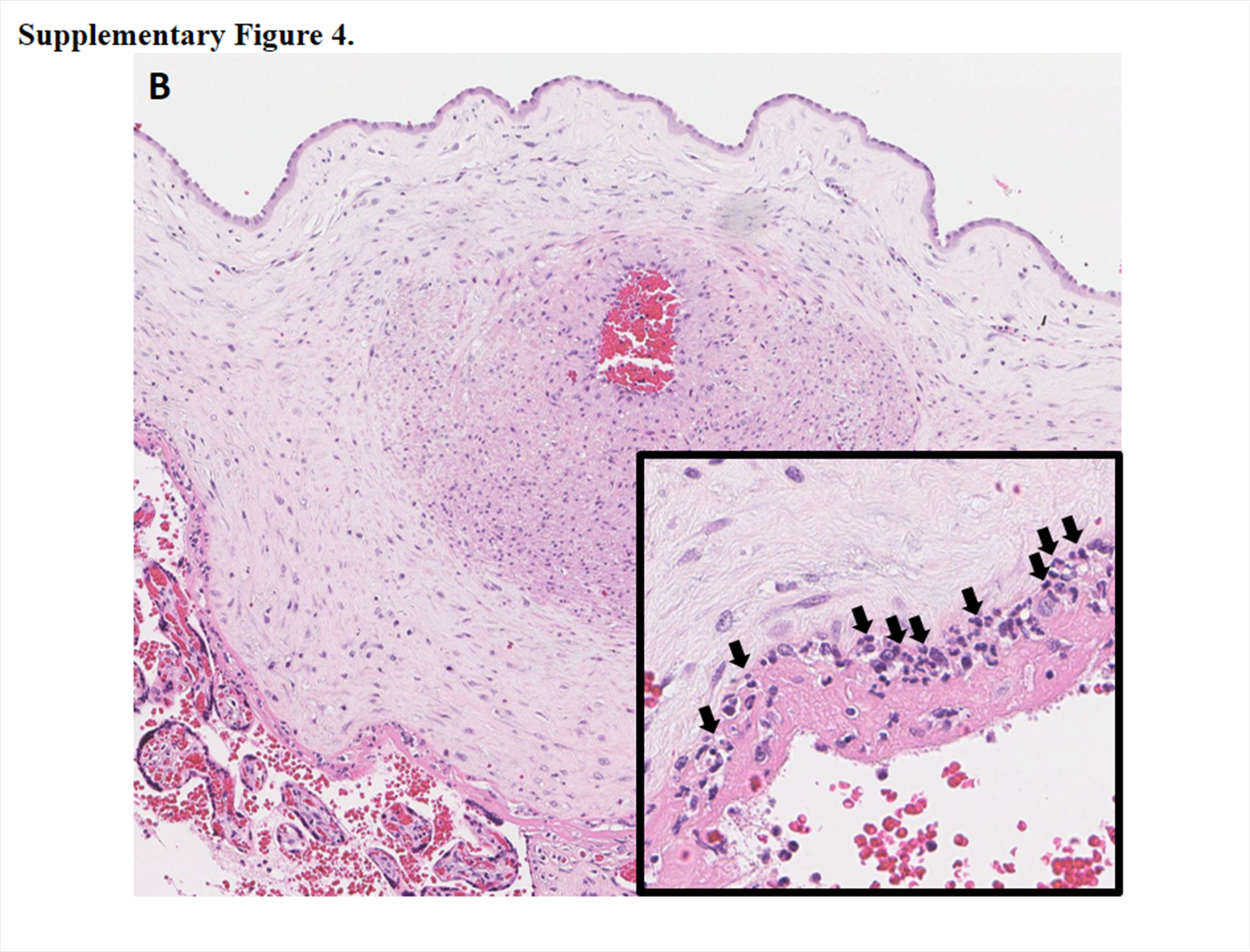

Supplement: Supplementary file 5 [file Image_5.TIF]

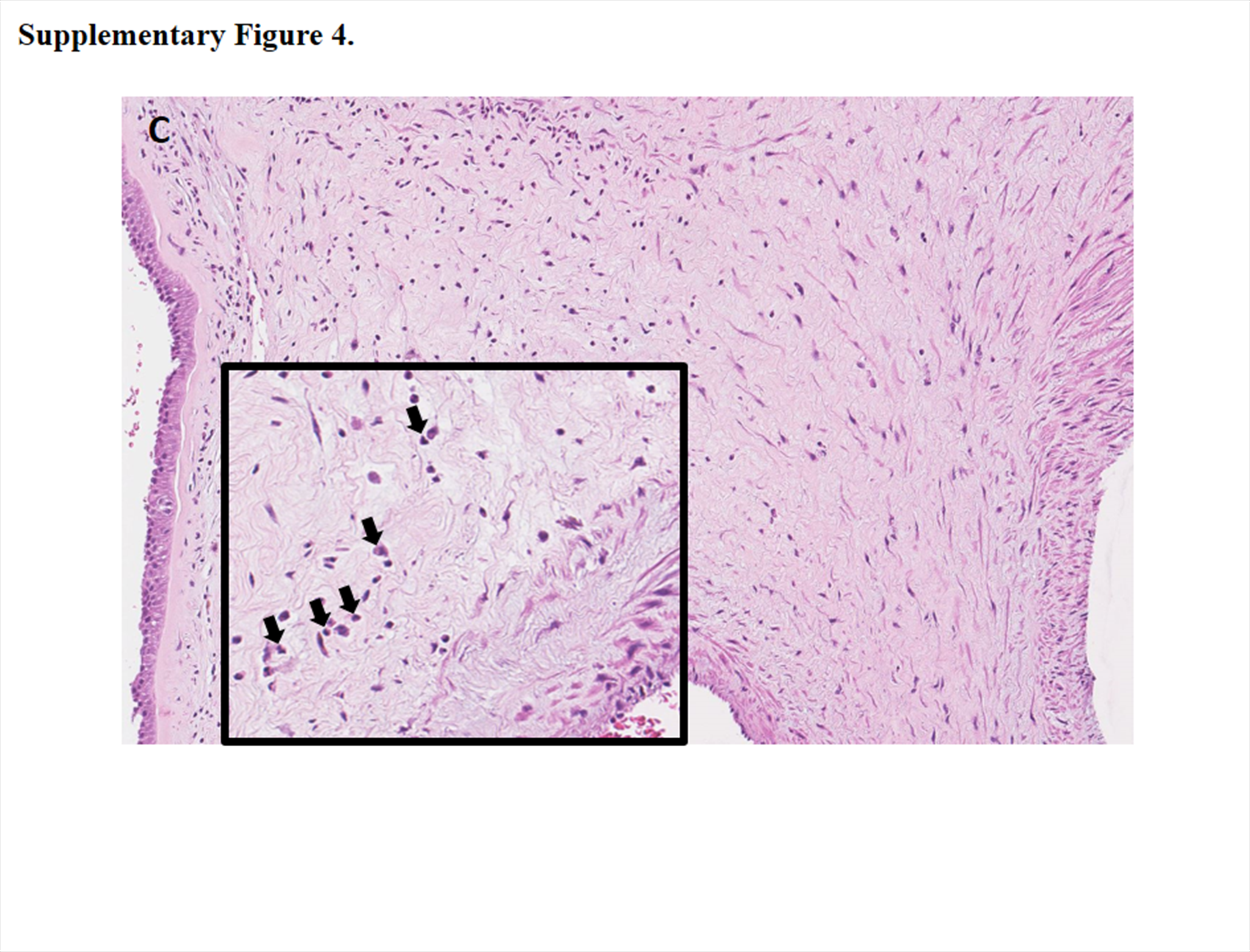

Supplement: Supplementary file 6 [file Image_6.TIF]

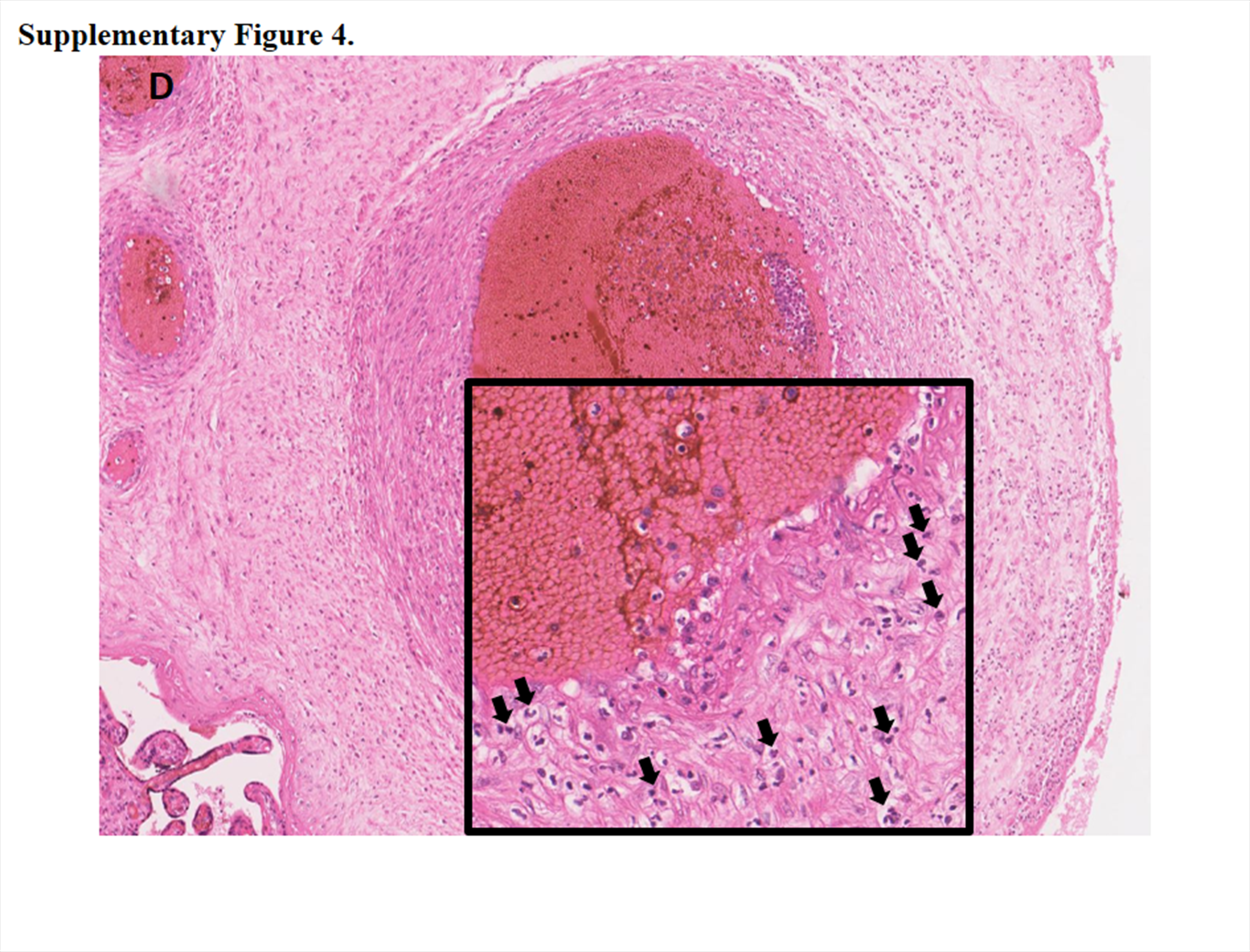

Supplement: Supplementary file 7 [file Image_7.TIF]
