# Supplementary material for: The Relationship Among Intra-Amniotic Inflammatory Response, The Progression of Inflammation in Chorionic Plate and Early-Onset Neonatal Sepsis
Source: Front Pediatr. 2021 Apr 29;9:582472. doi: 10.3389/fped.2021.582472 (PMC8116513; doi:10.3389/fped.2021.582472)
Supplement: Supplementary file 8 [file Data_Sheet_1.pdf]

Supplementary Material

Supplementary Tables

Supplementary Table 1. Previous studies examining the relationship between acute inflammation in chorionic plate (CP) as an independent compartment and amniotic fluid (AF) results.

**Primary author (year)<br><br>[Reference number]**	**Number<br><br>(CP inflammation[+]<br><br>vs.<br><br>CP<br><br>inflammation[-])**	**GA at delivery**	**Types of PTB**	**Inclusion criteria**	**Diagnostic criteria for inflammation in CP**	**Subdivisions of CP**	**GA at amniocentesis**	**Amniocentesis-to-delivery interval**	**Frequency of IA infection/inflammation (inflammation-free CP vs. inflammation in the subdivisions of CP)**	**Was IAIR or EONS continuously increased according to the progression of inflammation in either CP or the full-detailed subdivisions of CP?**		
										**IAIR and the progression of inflammation in CP (but not full-detailed subdivisions of CP)**	**IAIR and the progression of inflammation in the full-detailed subdivisions of CP**	**EONS and the progression of inflammation in the full-detailed subdivisions of CP**

Romero R., et al. (1992)[4]	74<br><br>(23 vs. 51)	PTB<br><br>(22~36 wks)	PTL		≥ 1 focus of at least 5 PMNs in the subdivisions of CP	(Based on reference [28])<br><br>Stage1: SCF, mild<br><br>Stage2: SCF, severe<br><br>Stage3: CT of CP<br><br>Stage4: CVs	22~36 wks	< 48 hrs	AF culture (+)<br><br>(Stage 0 [4%] vs.<br><br>Stage 1 [0%] vs.<br><br>Stage 2 [33%] vs.<br><br>Stage 3 [63%] vs.<br><br>Stage 4 [73%])	N/A	N/A	N/A
Cherouny PH., et al. (1993)[13]	38<br><br>(4 vs. 34)	PTB<br><br>(≤35 wks)	PTL	Oligohydramnios (-), placental abruption or previa (-), SGA (-)	Accumulation of neutrophils in the IVS below the CP	Stage1: Subchorionic IVS<br><br>Stage 2: Chorion<br><br>Stage 3: Amnion	≤ 34 wks	< 1 wks	N/A	Yes<br><br>(AF NAP-1/IL-8 level was higher in cases with stage 3 inflammation in CP than in cases with stage 1 inflammation in CP or stage 2 inflammation in CP.)	N/A	N/A
Yoon BH., et al. (1995)[14]	50<br><br>(38 vs. 12)	PTB<br><br>(24~35 wks)	PTL, preterm-PROM	Singleton,	>1 focus of at least 10 neutrophilic collections or diffuse inflammation in the subdivisions of CP	Stage 1: SCF<br><br>Stage 2: CT and/or CVs	23~35 weeks	< 72 hrs	N/A	N/A	N/A	N/A

Negishi H., et al. (1996)[15]	19<br><br>(12 vs. 7)	PTB	PTL	Singleton & twin	PMN infiltration in the subdivisions of CP	Stage 1: Subchorionic IVS<br><br>Stage 2: Chorion<br><br>Stage 3: Amnion	24~36 wks	< 24 hrs	AF culture (+)<br><br>(Stage 1 [0% (0/3)] vs.<br><br>Stage 2 [0% (0/0)]<br><br>vs.<br><br>Stage 3 [33% [1/3])	Yes<br><br>(AF IL-6 levels were continuously increased with the increasing stage of inflammation in CP)	N/A	N/A
Tsuda A., et al. (1998)[16]	110<br><br>(69 vs. 41)	Term birth & PTB	PTL	Singleton,	Neutrophils infiltration in the subdivisions of CP	Stage 1: Subchorionic IVS<br><br>Stage 2: Chorion<br><br>Stage 3: Amnion	16~31 weeks	N/A	N/A	Yes<br><br>(AF IL-6 level was associated with increasing stage of inflammation in CP)	N/A	N/A
Lee SD., et al. (2004)[17]	204<br><br>(180 vs. 24)	N/A	N/A	Singleton,<br><br>Intact membranes	>1 focus of at least 10 neutrophilic collections or diffuse inflammation in the subdivisions of CP	Stage 1: SCF<br><br>Stage 2: CT and/or CVs	N/A	< 72 hrs	N/A	N/A	N/A	N/A
Kidokoro K., et al. (200	60<br><br>(15 vs. 45)	PTB<br><br>(16~35 wks)	PTL or preterm-PROM	Singleton,	PMN infiltration in the subdivisions of CP	Stage 1: Subchorionic space<br><br>Stage 2: IVS	16~35 wks	< 48 hrs	N/A	Yes<br><br>(AF neutrophil elastase level was associated with	N/A	N/A

6)[18]						Stage 3: Amniotic cavity				increasing stage of inflammation in CP)		
Buhimschi IA., et al. (2008)[19]	158<br><br>(55 vs. 102)	Term birth & PTB<br><br>(17~40 wks)	PTL or preterm m-PROM	Singleton, anhydramnios (-)	Neutrophil infiltration in the subdivisions of CP	Stage 1: IVS<br><br>Stage 2: Chorion<br><br>Stage 3: Amnion	17~36weeks	N/A	IAI (MR score§ ≥1)<br><br>(Stage 0 [51%] vs.<br><br>Stage 1 [72%] vs.<br><br>Stage 2 [81%] vs.<br><br>Stage 3 [91%])	Yes<br><br>(The degree of IAIR [MR score§] was directly associated with the severity of inflammation in CP)	N/A	N/A
Kacerovsky M., et al. (2009)][20]	29<br><br>(24 vs. 5)	N/A	Preterm m-PROM	Singleton, AGA	Few PMNs in the subdivision of CP	Stage 1: CT of CP<br><br>Stage 2: CVs<br><br>* PMNs in SCF were not classified as inflammation in CP	24~36 wks	N/A	N/A	N/A	N/A	N/A
Miura H., et al. (2011)[21]	56<br><br>(7 vs. 47)	PTB (< 37 wks) or spontaneous abortion (< 22 wks)	PTL or preterm m-PROM		Neutrophil infiltrations in the subdivisions of CP	Stage 1: Subchorionic space<br><br>Stage 2: IVS<br><br>Stage 3:Amniotic cavity	15~35 wks	< 48 hrs	N/A	Yes<br><br>(Neutrophil elastase concentration was significantly higher in patients with stage 3 inflammation in CP	N/A	N/A

										than in patients with other stages of inflammation in CP.)		
Yoneda S., et al. (2015)[22]	428<br><br>(234 vs. 194)	PTB (50%) & term birth (50%)	PTL	Singleton, Intact membranes<br><br>-PTB: after 2 days of maternal steroid injection<br><br>-Term birth:<br><br>PROM (-) and<br><br>TOL (-)	Maternal neutrophils in the subdivision of CP	Stage 1: Between decidua and CP<br><br>Stage 2: CT of CP<br><br>Stage 3: Amnion	Intra-operative AF only	N/A	N/A	Yes<br><br>(The cutoff value of AF-IL-8 for predicting each stage of inflammation in CP increased with increasing stage of inflammation in CP.)	N/A	N/A
Dong YLZE., et al. (198	123<br><br>(35 vs.88)	Term birth (95.9%) & PTB (4.1%)	N/A	PROM for more than 10 hrs	Presence of PMNs in CP (placenta)	N/A	N/A<br><br>* AF obtained via transcervical	N/A	N/A<br><br>*Total colony count of bacteria in AF was strongly	N/A	N/A	N/A

7)[23]]		intrauterine aspiration (i.e., AF culture & total colony count of bacteria in AF)	associated with increasing grade, not progression, of inflammation in CP.

§ MR score was defined by the presence or absence of each of the four protein biomarkers such as neutrophil defensin 1 and 2, and calgranulin A and C.

AF, amniotic fluid; *BW*, birth-weight; *CA*, chorioamnionitis; *CP*, chorionic plate; *CT*, connective tissue; *CVs*, chorionic vessels; *GA*, gestational age; *IA*, intraamniotic; *IAIR*, intraamniotic inflammatory response; *IVS*, intervillous space; *LGA*, large for gestational age; *MFI*, maternal-fetal indication; *MR score*, Mass Restricted score; *N/A*, not available; *preterm-PROM*, preterm premature rupture of membranes; *PROM*, premature rupture of membranes; *PTB*, preterm birth; *PTL*, preterm labor and intact membranes; *SCF*, subchorionic fibrin; *SGA*, small for gestational age

Supplementary Figure legends

Supplementary Figure 1. Flow chart of the study population.

Supplementary Figure 2. The frequency of suspected or proven early-onset neonatal sepsis (EONS) or immediately neonatal death shortly after birth [A], proven EONS or immediately neonatal death shortly after birth [B] and immediately neonatal death shortly after birth [C] according to the progression of inflammation within chorionic plate (CP) (i.e., stage-0, inflammation-free CP vs. stage-1, inflammation restricted to subchorionic fibrin [SCF] vs. stage-2, inflammation in the connective tissue [CT] of CP without chorionic vasculitis vs. stage-3, chorionic vasculitis) among preterm gestations. Each P value is shown in the graph. Ten neonates were excluded from these analyses among 309 cases because they had no information about EONS in the medical records (n=10) and thus could not be evaluated with respect to the presence or absence of EONS, or immediately neonatal death shortly after birth.

Supplementary Figure 3. Plot of predicted probability of the occurrence of suspected or proven EONS, or immediately neonatal death shortly after delivery according to gestational age (GA) at delivery and the presence or absence of inflammation in chorionic plate (CP) among 302 cases with GA at delivery (≥ 24 weeks) of study population. Inflammation in CP was associated with an increase in suspected or proven EONS, or immediately neonatal death shortly after delivery after adjusting for GA at delivery (Odds ratio [OR]=5.887, 95% confidence interval [CI] 2.448-14.155, P=0.000075). Among 302 cases with GA at delivery (≥ 24 weeks), nine neonates were excluded from these

analyses because they had no information about EONS in the medical records (n=9) and thus could not be evaluated with respect to the presence or absence of suspected or proven EONS, or immediately neonatal death shortly after birth.

Supplementary Figure 4. Histopathology of the progression of inflammation in chorionic plate (CP). Hematoxylin and eosin stained histologic sections of CP are shown as follows: inflammation-free CP (A), inflammation restricted to subchorionic fibrin (SCF) (B), inflammation in the connective tissue (CT) of CP without chorionic vasculitis (C), and chorionic vasculitis (D). These images are based on the magnification setting ×80, and the insets of panels are based on the magnification setting ×200. A lot of neutrophils (arrows) are shown in supplementary Figure 4 B, C and D.
